# A chemo-enzymatic oxidation cascade to activate C–H bonds with in situ generated H_2_O_2_

**DOI:** 10.1038/s41467-019-12120-w

**Published:** 2019-09-13

**Authors:** Simon J. Freakley, Svenja Kochius, Jacqueline van Marwijk, Caryn Fenner, Richard J. Lewis, Kai Baldenius, Sarel S. Marais, Diederik J. Opperman, Susan T. L. Harrison, Miguel Alcalde, Martha S. Smit, Graham J. Hutchings

**Affiliations:** 1 Cardiff Catalysis Institute, School of Chemistry, Cardiff University, Main Building, Park Place, Cardiff, CF10 3AT UK; 2 Department of Chemistry, University of Bath, Claverton Down Bath, BA2 7AY UK; 3 Department of Microbial, Biochemical and Food Biotechnology, University of the Free State, Bloemfontein, South Africa; 4 South African DST-NRF Centre of Excellence in Catalysis, C*Change, University of Cape Town, Private Bag, Rondebosch, 7701 Cape Town, South Africa; 5 Centre for Bioprocess Engineering Research (CeBER), Department of Chemical Engineering, University of Cape Town, Private Bag X3, Rondebosch 7701 Cape Town, South Africa; 6 BASF SE, RBW/OS - A 30, Carl-Bosch-Strasse 38, 67056 Ludwigshafen am Rhein, Germany; 7 Department of Biocatalysis, Institute of Catalysis, CSIC, 28049 Madrid, Spain

**Keywords:** Asymmetric catalysis, Heterogeneous catalysis, Chemical engineering

## Abstract

Continuous low-level supply or in situ generation of hydrogen peroxide (H_2_O_2_) is essential for the stability of unspecific peroxygenases, which are deemed ideal biocatalysts for the selective activation of C–H bonds. To envisage potential large scale applications of combined catalytic systems the reactions need to be simple, efficient and produce minimal by-products. We show that gold-palladium nanoparticles supported on TiO_2_ or carbon have sufficient activity at ambient temperature and pressure to generate H_2_O_2_ from H_2_ and O_2_ and supply the oxidant to the engineered unspecific heme-thiolate peroxygenase PaDa-I. This tandem catalyst combination facilitates efficient oxidation of a range of C-H bonds to hydroxylated products in one reaction vessel with only water as a by-product under conditions that could be easily scaled.

## Abstract (web summary)

Continuous low-level supply or in situ generation of hydrogen peroxide (H_2_O_2_) is essential for the stability of unspecific peroxygenases. Here, the authors demonstrate that AuPd / TiO_2_ can generate sufficient H_2_O_2_ for the engineered unspecific heme-thiolate peroxygenase PaDa-I to oxidise a range of C-H bonds.

## Introduction

Selective oxidative CH-activation of hydrocarbon substrates remains a challenge for both heterogeneous catalysis and biotechnology. Many potential heterogeneous catalysts suffer from low regioselectivity and are prone to overoxidation of the substrate^1–3^. For example, the highly optimized commercial oxidation of cyclohexane typically produces cyclohexanol and cyclohexanone, which can be utilized as feedstocks for the production of adipic acid and caprolactam, together with considerable amounts of overoxidation products^4^. In order to prevent diminishing yield due to overoxidation, conversion of cyclohexane has to be kept as low as 6%, leading to poor space-time yields and high recycling efforts. The radical reaction pathway of this oxidation cannot be manipulated to produce the alcohol selectively^5,6^.

A number of enzymes have been characterized that do not suffer these shortcomings^7^. Among these are unspecific peroxygenases (UPOs), extracellular fungal heme-thiolate enzymes that only use hydrogen peroxide (H_2_O_2_) as both oxygen donor and final electron acceptor to generate the activated oxygen species at the active site (reactive Compound I)^8,9^. Peroxygenases have been described as ideal catalysts for CH-activation because they are relatively stable with high efficiency toward H_2_O_2_ and in addition do not, like P450 monooxygenases, require ancillary flavoproteins and cofactors that need regeneration^9,10^. However, a limitation to scale up of UPO based chemical transformations is that they can be easily deactivated by modest concentrations of H_2_O_2_, thus requiring a constant controlled supply of H_2_O_2_ at low concentrations^8^. This makes the in situ production of low (mM) levels of H_2_O_2_ practically the most efficient way to carry out the transformation without the need to add large volumes of prepared commercial H_2_O_2_ solutions, which typically contain 50–70 wt% H_2_O_2_.

Accordingly, several systems have been developed to supply H_2_O_2_ to UPOs and the closely related chloroperoxidase from *Caldariomyces fumago* (CPO) used for sulfoxidation and hydroxylation of benzylic carbons. However, to date no system has been simple and robust enough to allow large scale applications^11–13^. The generation of H_2_O_2_ with enzymes such as glucose oxidase (GOX) requires co-substrates that are transformed to side products (in the case of glucose oxidase, gluconic acid) and as such produces a complex reaction mixture which is problematic. The GOX system is very (atom-)inefficient as only two of a potential 24 electrons are used to generate one H_2_O_2_ equivalent. Additionally, the production of gluconic acid in the same reaction vessel as the UPOs necessitates continuous pH control to maintain the activity of the UPOs. A recently described formate oxidase, *Ao*FOx from *Aspergillus oryzae*, which uses sodium formate as source of reducing equivalents to produce H_2_O_2_, promises a significant improvement on GOX in terms of side product production, but still requires acid titration to maintain a constant pH^14^.

As an alternative, Hollmann and coworkers used the UPO from *Agrocybe aegerita* (*Aae*UPO) together with an oxidase/dehydrogenase cascade consisting of five enzymes to convert methanol to carbon dioxide, avoiding the accumulation of side products such as formic acid or formaldehyde from methanol oxidation^15^. Although they obtained high total turnover numbers (TTNs) for the hydroxylation of ethylbenzene (294,700 mol mol^−1^), the system still required the addition of nicotinamide adenine dinucleotide (NAD^+^) as a cofactor, and a catalytic system requiring five different enzymes will only be economically feasible for production of high-value products due to the complexity of controlling the reaction and inhibition kinetics of five enzymatic processes operating in parallel^15^. In the same direction, a bienzymatic cascade has recently been applied for the in situ generation of two equivalents of H_2_O_2_ from methanol, releasing formate as a byproduct, during the production of human drug metabolites, by an evolved *Aae*UPO mutant^16^. The complex photobiocatalytic system Churakova et al. used with native *Aae*UPO for the hydroxylation of several substrates including ethylbenzene, cyclohexane and octane also still required flavin mononucleotide as a cofactor (FMN) and co-substrate (ethylenediaminetetraacetic acid) to supply reducing equivalents^17^. Further recent papers by Hollmann and coworkers describe in situ H_2_O_2_ production by photocatalytic oxidation of methanol and water using various gold loaded TiO_2_ photocatalysts^18–20^. When using recombinant *Aae*UPO (r*Aae*UPO) and methanol as electron donor they obtained TTNs in the order of 80,000 with ethylbenzene and 61,500 with cyclohexane. TTNs were about 50% lower when water was used as electron donor. However, the scale up of photocatalytic transformations is challenging and costly. Exposure of the enzyme to the photocatalyst was reported to cause significant loss in enzyme activity due to oxidative damage by H_2_O_2_ decomposition and reactive oxygen species being generated under light irradiation^18–20^. This necessitated immobilization of the enzyme onto a solid carrier to minimize exposure to the photocatalytic TiO_2_ surface and increase lifetime. Horst et al. also obtained a very high TTN of 400,000 for the hydroxylation of ethylbenzene with an electroenzymatic system using r*Aae*UPO and a gas-diffusion cathode that contained a H_2_O_2_ generating catalyst^21^. This latter system demonstrated the potential of the UPOs for industrial application if H_2_O_2_ is generated in situ, although enzyme lifetime was linked to current density applied in the electrochemical cell, possibly due to the high H_2_O_2_ concentrations at the electrode surface resulting in a significant concentration gradient in the system and enzyme degradation due to localised high H_2_O_2_ concentrations.

A simple alternative to the enzymatic, photocatalytic and electrochemical methods described above would be to directly generate H_2_O_2_ from H_2_ and O_2_ with supported gold-palladium (AuPd) heterogeneous catalysts, which have been shown to be able to achieve H_2_ selectivity of >95% towards H_2_O_2_ making an atom efficient process possible and would represent an approach that does not require any significant re-design of current catalytic reactors to include light or electrical energy input^22,23^. Continuous H_2_O_2_ generation from H_2_ and O_2_ by AuPd catalysts generally requires high pressures and subambient temperatures and often also the addition of acid or halide promoters, conditions that are not ideal for enzymes^24,25^. Hence, a reaction conditions gap exists to couple the heterogeneous catalytic system with the current operational window of UPOs. Indeed, for H_2_O_2_ synthesis, temperatures are typically subambient (e.g. 2 °C), gas pressures are high (40 bar), and pH is low pH (<3), whereas for UPO activity, preferred conditions are ambient temperature and pressure and pH 6–7. Karmee et al. have nevertheless demonstrated the sulfoxidation of thioanisole by CPO in reactions using Pd/C for H_2_O_2_ generation at high pressure (130 bar) and 40 °C in supercritical carbon dioxide/water, which relies on setting up a biphasic scCO_2_/H_2_O phase separating the H_2_O_2_ synthesis, which occurs in the scCO_2_ phase and the biocatalysis, which occurs in the aqueous phase^11^.

We recently demonstrated AuPd bimetallic catalysts can produce H_2_O_2_ in water at ambient temperature and 40 bar working pressure^26^. The question arose whether this class of catalysts can be used to produce H_2_O_2_ to cross the conditions gap for a UPO catalyzed reaction at both ambient temperature and low pressure in phosphate buffered solution. Compared to the other solutions of in situ H_2_O_2_ formation this approach would provide an atom efficient reaction, using H_2_ as the reductant rather than glucose in the case of the GOX approach resulting in much simpler reaction mixtures - as water is the only byproduct - requiring no extra pH control due to the presence of byproducts of other enzymatic cascade processes. In contrast to photocatalytic methods using UV light the unselective production of reactive oxidative species could be minimized, increasing UPO stability. In addition the simplicity of the proposed reaction combination in contrast to other catalytic approaches makes this approach more easily scalable into existing chemical infrastructure, requiring only the use of common gas-liquid-solid stirred tank reactors rather than the design of new photo or electrochemical reactors or the incorporation of supercritical fluids, and requires only H_2_ and air as the feed—greatly reducing the complexity of H_2_O_2_ delivery compared to current approaches.

**Fig. 1:**
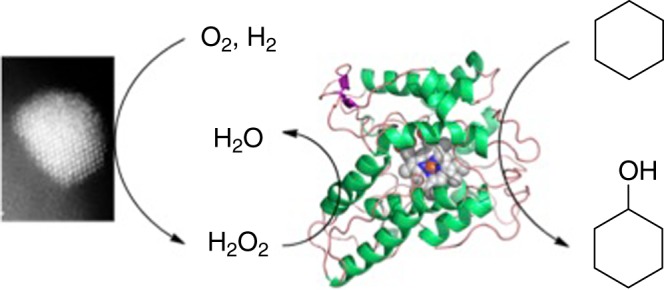
Proposed reaction system. A combination of heterogeneous catalysis and biocatalysis can be used to oxidize cyclohexane using in situ produced H_2_O_2_

Here we show this is indeed possible and that a tandem catalyst system formed by AuPd/TiO_2_ for the in situ gradual supply of H_2_O_2_ and the evolved *Aae*UPO (referred to as PaDa-I) can facilitate hydroxylation of a number of truly unactivated sp^3^ carbons such as cyclohexane and ethylbenzene (Fig. 1) at mild conditions^27–29^.

## Results

### Compatibility of the heterogeneous and enzyme catalyst

**Fig. 2:**
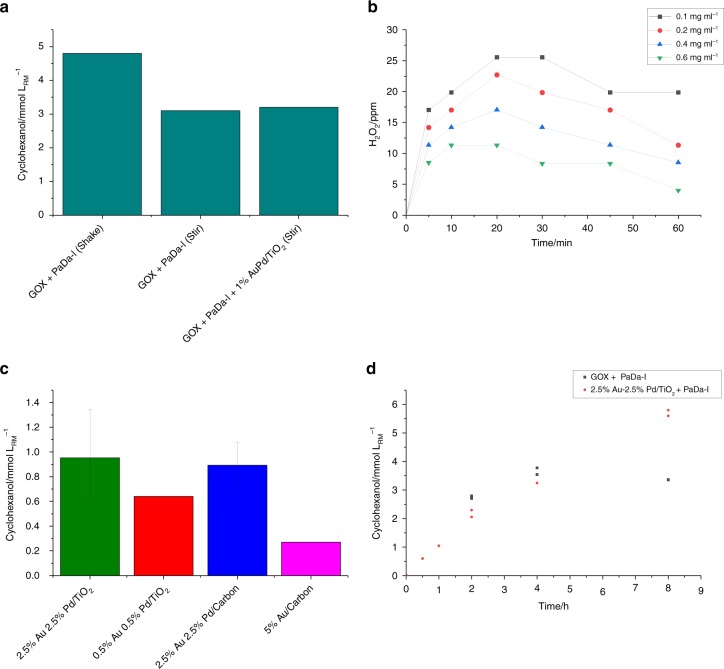
Feasibility of Coupling Catalytic Systems. **a** Cyclohexanol production in control reactions to determine the effect of the metal catalyst on unspecific peroxygenase activity when H_2_O_2_ is generated using GOX. **b** H_2_O_2_ synthesis with various catalyst concentrations under flowing (30 ml min^−1^) 80% H_2_ in air. Reaction Conditions: Solvent (H_2_O) 50 ml, ambient temperature and pressure, gas flow 30 ml min^−1^, 500 rpm stirring. Errors associated in the measurement of H_2_O_2_ by titration were ± 3 ppm. **c** Cyclohexane conversion to cyclohexanol using PaDa-I (15 U ml_RM_^−1^) in potassium phosphate buffer (100 mM, pH 6) and H_2_O_2_ generated in situ by metal catalysts. Efficiency of different AuPd catalysts (0.5 mg ml^−1^) in the presence of 77% H_2_ in air. **d** Time course results for cyclohexanol formation by PaDa-I (15 U ml_RM_^−1^) was coupled with in situ H_2_O_2_ generation by 2.5% Au-2.5% Pd/TiO_2_ (0.1 mg ml_RM_^−1^) using a gas mixture of 77% H_2_ in air (solid diamonds) or by GOX (0.2 U ml_RM_^−1^) using 200 mM glucose and air (circles). The GOX reactions were carried out using the same experimental setup as the metal catalyst reactions. Typically analysis of products was ± 0.2 mM as determined by GC (on duplicate experiments)

Initial control reactions using GOX to generate H_2_O_2_ demonstrated that cyclohexane hydroxylation reactions to produce cyclohexanol could be carried out either using shaking or stirring to facilitate mixing. In addition, these reactions showed that the 0.5 wt% Au–0.5 wt% Pd/TiO_2_ (1 mg ml^−1^) catalyst had no negative effect on the PaDa-I reaction when GOX was used to produce H_2_O_2_ (Fig. 2a); demonstrating that the PaDa-I could still operate in the presence of the metal catalyst. Considering H_2_O_2_ production requires H_2_ and O_2_ mixtures, which are combustible between H_2_ concentrations of 5 and 75%, H_2_O_2_ synthesis experiments were carried out in an open system by flowing 50 ml min^−1^ of gas (2%, 80%, 90% H_2_ in air) through an aqueous solution of metal catalyst (0.1 mg ml^−1^). With 80% H_2_ in air, a peak concentration of 25 ppm H_2_O_2_ (0.74 mM) was produced (Supplementary Fig. 1). The effect of the catalyst concentration was also investigated between 0.1 and 0.6 mg ml^−1^ (Fig. 2b). At higher catalyst loadings, the maximum amount of H_2_O_2_ generated decreased while at lower loadings, the peak concentration was higher and the system was capable of producing a sustained concentration of H_2_O_2_. These results were promising, given that Molina-Espeja et al. had reported the Michaelis constant (*K*_m_) of PaDa-I for H_2_O_2_ at pH 7 in 100 mM potassium phosphate buffer as 0.49 mM (17 ppm) when it was produced by *Saccharomyces cerevisiae*^27^ and 1.53 mM (53 ppm) when produced by *Pichia pastoris*^28^. Thus, concentrations of 1 or 2 mM H_2_O_2_ (34 or 68 ppm) were used successfully in enzyme activity experiments^27,28^. These concentrations compared with our results suggested that the AuPd catalyst would be active enough at ambient conditions to supply H_2_O_2_ in order for the PaDa-I to carry out the oxidation of cyclohexane at significantly lower precious metal catalyst loadings than previous studies^19^.

Four different AuPd catalysts, namely 2.5 wt% Au–2.5 wt% Pd/TiO_2_^29^, 0.5 wt% Au–0.5 wt% Pd/TiO_2_^23^, 2.5 wt% Au–2.5 wt% Pd on carbon and 5 wt% Au on carbon^30^ (0.5 mg ml_RM_^−1^) were tested for their ability to generate H_2_O_2_ for PaDa-I conversion of cyclohexane with 77% H_2_ in air (15 U ml_RM_^−1^, enzyme activities were determined using ABTS and H_2_O_2_ as substrate with one unit defined as the amount of enzyme needed to converted 1 µmol in 1 min) (Fig. 2c). All of the AuPd catalysts gave promising results with between 0.6 and 1.3 mmol cyclohexanol l_RM_^−1^ produced after 2 h with these catalysts generating H_2_O_2_. In the absence of metal catalyst, negligible cyclohexanol (less than 0.03 mM) was detected when 77% H_2_ in air was used (Supplementary Table 1). In all cases the presence of cyclohexanone resulting from overoxidation of cyclohexanol was observed only in negligible amounts in the gas chromatograms after 2 and 4 h, indicating that presence of the metal catalyst does not result in significant overoxidation (Supplementary Fig. 2).

A set of reactions with a maximum reaction time of 8 h indicated that the tandem reaction system was relatively stable with a maximum of 5.7 mmol cyclohexanol l_RM_^−1^ produced in 8 h (Fig. 2d). The reactions in which 0.2 U ml_RM_^−1^ GOX was used to produce H_2_O_2_ gave comparable cyclohexanol production after 2 h and 4 h, but then leveled off to give only 3.4 mmol cyclohexanol l_RM_^−1^ after 8 h. This time course most likely resulted from the operational instability of GOX caused by inactivation by H_2_O_2_ as well as auto-inactivation but demonstrates that the initial rate of the tandem process is the same for both systems^31^. The total turnover number (TTN) of 16,000 calculated for the 8 h reactions with the 2.5 wt% Au–2.5 wt% Pd/TiO_2_ catalyst supplying H_2_O_2_ is in the same order as the TTN of 17 900 reported for cyclohexane hydroxylation by wild-type *Aae*UPO with H_2_O_2_ generated by photocatalysis (reaction time 5 h)^17^. Assays of residual activity remaining in the enzyme after 4 h of reaction in the GOX and metal catalyst systems showed that in both cases the enzyme retained 50–55% of the initial activity. Hence, the H_2_O_2_ producing metal catalyst is not accelerating the deactivation of the PaDa-I during extended reaction times.

### System optimisation and substrate scope

**Fig. 3:**
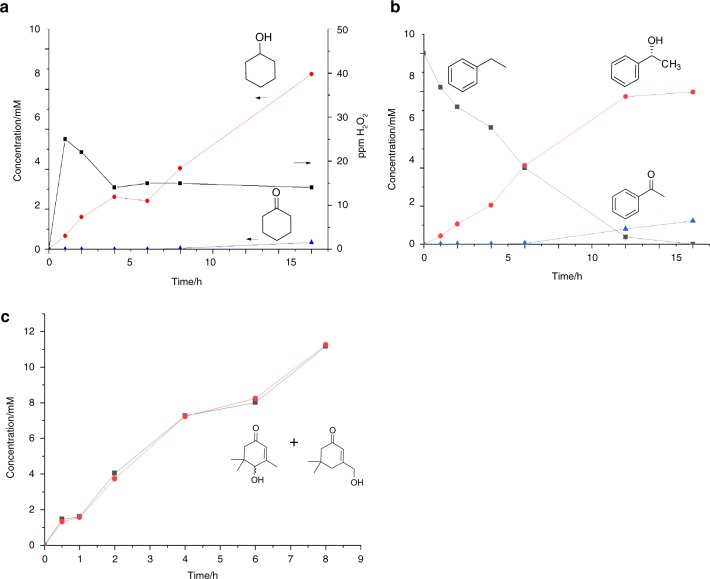
Hydroxylation reactions using tandem catalysis system. Time on line hydroxylation reactions of **a** H_2_O_2_ production (black squares) (in the absence of enzyme) and cyclohexane (10 mM) oxidation to cyclohexanol and cyclohexanone **b** Ethylbenzene (10 mM) oxidation to 1-phenylethanol **c** Isophorone (30 mM) oxidation to 4 and 7-hydroxyisophorone when PaDa-I (15 U ml_RM_^−1^) was coupled with in situ H_2_O_2_ generation by 0.5% Au-0.5% Pd/TiO_2_ (0.1 mg ml_RM_^−1^) using a gas mixture of 80% H_2_ in air in a sealed system under 2 bar total pressure

Due to the volatile nature of substrates such as cyclohexane, a flowing system with gas bubbling is not optimal and relies on constant substrate addition to prevent complete depletion of the substrate. Following the feasibility experiments showing that combining the two catalytic systems was possible, reactions were carried out in sealed pressurized glassware with a range of substrates. Into the reactor was placed the substrate (10 mM), and the tandem catalyst system formed by the heterogeneous catalyst and the biocatalyst in buffered reaction media. All reactions were carried out with the same initial substrate and enzyme concentration limiting the TTN to ~30,000 in these studies. The system was pressurized to 2 bar under a pre-mixed gas mixture of 80% H_2_ and 20% air and stirred at 250 rpm. Under these conditions H_2_O_2_ was produced over time (Fig. 3a) in a similar range 15–25 ppm to the flowing system over extended reaction times despite the lack of gas bubbling. The results in Fig. 3a demonstrate that under these conditions cyclohexane hydroxylation can be carried out to produce cyclohexanol with high yields (87%) and TTN of 25,300 with negligible overoxidation to cyclohexanone indicating that the metal H_2_O_2_ producing catalyst is not facilitating further overoxidation reactions as observed in our previous experiments using the gas flow reactor.

The ability of biocatalysts to carry out chiral transformations is a major advantage over traditional heterogeneous systems. The hydroxylation of ethylbenzene was carried out over extended times with near full conversion being reached at 16 h (Fig. 3b). While in this case some overoxidation was observed when the reaction had consumed the majority of ethylbenzene, again a high TTN was observed (25,900) at 12 h with near complete selectivity to 1-phenylethanol. Chiral analysis of the 1-phenylethanol produced showed an e.e. of ~98% towards R-1-phenylethanol at 16 h, which is comparable to reported values concerning the peroxygenase alone indicating that the presence of the metal catalyst does not affect the stereoselectivity of the PaDa-I hydroxylation reactions (Supplementary Fig. 3)^19^. To demonstrate the possibility of achieving higher TTN a reaction was carried out with 20 mM starting concentration for 16 h resulting in 13.1 mM of 1-phenyl ethanol and 2.9 mM of acetophenone at a TTN of 51,400. To test the system at extended reaction times an additional reaction was carried out with an initial concentration of 30 mM ethylbenzene at the same metal and enzyme catalyst loadings. After an initial 16 h of reaction, a further 30 mM of substrate was added and gas re-charged and this was repeated after another 24 h - resulting in 90 mM of substrate addition over 64 h. The system was able to produce 46 mM of 1-phenyl ethanol and 14 mM of acetophenone - achieving a TTN of 201,000 showing that this is among the most efficient tandem heterogeneous H_2_O_2_ delivery—UPO systems reported to date.

The selective functionalization of isophorone provides a route to synthetic precursors of both fragrance molecules and the building blocks of carotenoids and tocopherols (Vitamin E)^32,33^. α-Isophorone is produced industrially on a large scale and selective hydroxylation to 4-hydroxyisophorone is a target transformation to produce ketoisophorone without the industrially executed detour of first isomerizing to ß-isophorone^34–36^. The direct transformations have been reported using both homogeneous metal chlorides as catalysts at 80 °C in the presence of radical initiators and biocatalytic transformations based on P450s requiring cofactors^37,38^. As a proof of principle using our tandem catalyst system we were able to drive the hydroxylation of isophorone (30 mM) to produce a mixture of 4-hydxoyisophorone and 7-hydroxyisophorone in roughly equal amounts over a 6 h period at a combined yield of 75% (Fig. 3c). The unselective nature of the hydroxylation is inherent to the selectivity of the enzyme but acts as a demonstration that this transformation could be possible with a more selective peroxygenase, which could be engineered by new rounds of laboratory evolution. In all cases cyclohexane, ethylbenzene and isophorone reactions containing the metal catalyst producing H_2_O_2_ without the presence of the PaDa-I did not produce any hydroxylated products resulting from C–H activation as a result of the heterogeneous catalyst or the presence of H_2_O_2_ alone (Supplementary Fig. 4). In addition, the activity of the metal catalyst producing H_2_O_2_ alone towards the oxidation of cyclohexanol and 1-phenylethanol to the respective ketones was minimal (Supplementary Fig. 5).

The substrate scope was increased to include a wide range of C–H functionalization and is shown in Table 1 for a series of unoptimized reactions. Again, in the case of tetralin and propylbenzene negligible C-H activation was observed in the presence of the metal catalyst generating H_2_O_2_ without the PaDa-I (Supplementary Fig. 6). Oxygenated products were produced by the tandem catalyst system for this wide range of substrates and with high enantiomeric excess (>99%), (Supplementary Fig. 7) typical of the reaction previously carried out with the PaDa-I alone, demonstrating that the system does not change the specificity of the biocatalyst but simply provides a continuous feed of low-level H_2_O_2_ to facilitate clean hydroxylation. Reactions of styrene with the tandem catalysis system act as a demonstration of more complicated cascades that can be built by combing the two catalyst systems (Supplementary Fig. 8a). The AuPd catalyst used has no activity towards styrene epoxidation but is able to reduce styrene to ethylbenzene at these conditions (Supplementary Fig. 8b) leading to R-1-phenylethanol production with high enantiomeric excess (Supplementary Fig. 8c) through enzymatic hydroxylation along with styrene oxide. This reaction network of coupled C=C bond reduction and subsequent hydroxylation could be exploited to produce chiral alcohols from alkenes or mixtures of epoxides and chiral alcohols with proportions controlled by the design of the heterogeneous catalyst or reaction conditions. As with many process involving H_2_O_2_ in situ reactions H_2_ selectivity is challenging. The reported cyclohexane hydroxylation H_2_ to cyclohexanol selectivity is of the order of 10% (Supplementary Discussion 1); however, we have previously shown that high selectivity can be engineered through effective catalyst design^30,39^ and detailed understanding of the reaction kinetics and will be a key feature of any process intensification.

**Table 1:**
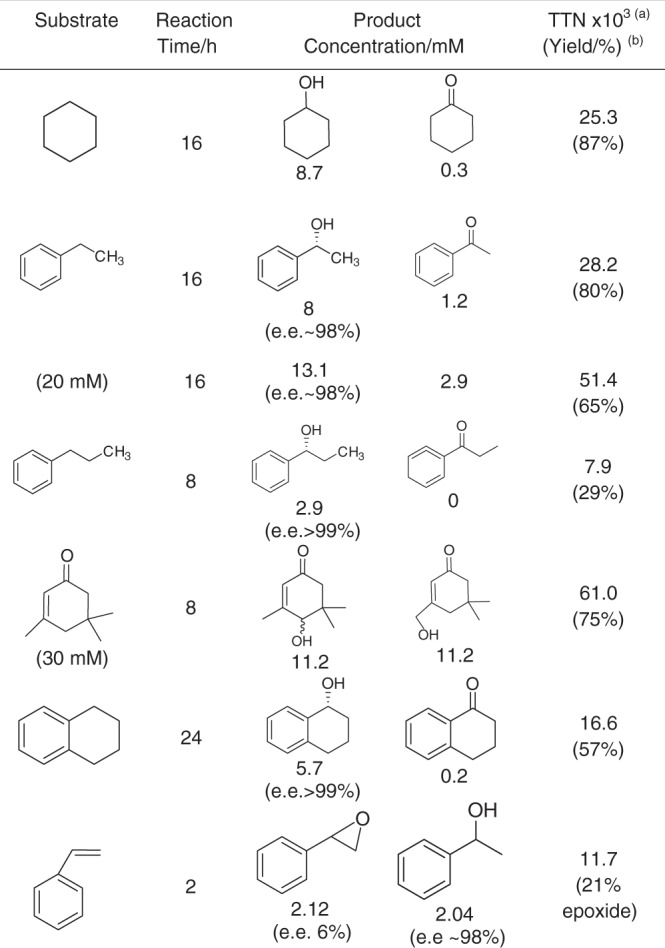
Substrate scope of tandem heterogeneous and biocatalytic system

PaDa-I (15 U ml_RM_^−1^) was coupled with in situ H_2_O_2_ generation by 0.5% Au-0.5% Pd/TiO_2_ (0.1 mg ml_RM_^−1^) using a gas mixture of 80% H_2_ in air in a sealed system under 2 bar total pressue. Substrate concentration was 10 mM unless otherwise stated

^a^TTN of C-H activation reaction accounting for secondary oxidation products

^b^To major (primary) hydroxylated product

The results reported here demonstrate the compatibility of AuPd chemo catalysts with the recently discovered UPOs to be used for hydroxylation of unactivated sp^3^ carbons through the in situ generation of H_2_O_2_. In this case we have demonstrated the potential for reactions that are operated commercially at a range of scales from commodity to fine chemicals. We consider that this chemo-enzymatic approach has potential for a wide range of applications in tandem reactions due to the ease of adaptation into current reactor technology and infrastructure, lower energy input as there is not requirement for illumination compared to recent photocatalytic approaches and inherent efficiency of the process in only producing water as a byproduct without the requirement for organic molecules such as MeOH as a supply of electrons. The nature of the catalyst materials as both effective hydrogenation and oxidation catalysts significantly extends the scope of the reaction cascades that can be considered and can open the pathways to efficient tandem heterogeneous/biocatalytic processes.

## Methods

### Catalyst preparation

A typical preparation of 1% AuPd/TiO_2_ was carried out according to the following procedure^23^. The requisite amounts of HAuCl_4_·3H_2_O solution (Sigma–Aldrich, 12.25 mg ml^−1^) and PdCl_2/_HCl solution (Sigma–Aldrich, 6 mg ml^−1^; HCl concentration: 0.58 M) were mixed in a 50 ml round-bottom flask, the volume of the solution was adjusted using deionized water to a total volume of 16 ml and immersed into an oil bath on a magnetic stirrer hot plate. The solution was stirred at 1000 rpm and the temperature of the oil bath was raised from room temperature to 60 °C over a period of 10 min. At 60 °C, metal oxide support material [1.98 g TiO_2_ (Degussa Evonik P25)] was added slowly over a period of 8–10 min with constant stirring. The subsequent slurry was stirred at 60 °C for an additional 15 min. Following this, the temperature of the oil bath was raised to 95 °C for 16 h leaving a dry solid. The solid powder was ground thoroughly. 400 mg of the sample was reduced at 10 °C min^−1^ under a steady flow of gas (5% H_2_/Ar). 2.5% Au-2.5% Pd/TiO_2_^29^, 2.5% Au-2.5% Pd/C and 5% Au/C^30^ were prepared by wet impregnation methods followed by calcination at 400 °C for 3 h.

### Unspecific peroxygenase preparation

Evolved *Aae*UPO (PaDa-I variant) designed in *Saccharomyces cerevisiae* was over produced in *Pichia pastoris* in a bioreactor and purified to homogeneity (Reinheitszahl value [Rz] [A_418_/A_280_] ∼ 2.4)^27,28^. Enzyme activities was determined using ABTS as substrate. Reactions were done in triplicate. 20 μl PaDa-I was added to 180 μl ABTS reaction mixture (100 mM sodium citrate–phosphate pH 4.4 with 0.3 mM ABTS and 2 mM H_2_O_2_) and substrate conversion was followed by measuring the absorption at 418 nm (ε 418 = 36000 M^−1^ cm^−1^). The PaDa-I concentration was appropriately diluted to give rise to linear enzyme kinetics. One unit is defined as the amount of enzyme that convert 1 µmol of substrate in 1 min.

### H_2_O_2_ synthesis testing in flowing system

H_2_O_2_ synthesis activity was evaluated at ambient temperature and pressure using a 200 ml dreschel bottle. 50 ml 0.1 M potassium phosphate buffer pH 6 was added containing catalyst concentrations ranging from 0.1 to 1 mg ml^−1^. During stirring using a magnetic stirrer, H_2_ in air mixtures were bubbled (30 ml min^−1^) through the bottle at compositions ranging from 2 to 90% by vol H_2_ controlled by a Brooks Mass Flow Controllers (flammability region of H_2_ in air 5–75 vol%). At periodic intervals aliquots were taken and the concentration of H_2_O_2_ was determined by titration against an acidified dilute Ce(SO_4_) solution using ferroin as an indicator.

### Cyclohexane oxidation testing

mmol cyclohexanol l_RM_^−1^ refers to mmol cyclohexanol produced per unit volume of the aqueous phase. Under some conditions the amounts of cyclohexane exceed the solubility limits resulting in two phases being present.

### Reactions using glucose oxidase for in situ H_2_O_2_ production

Biotransformations using an enzyme driven system for the in situ production of H_2_O_2_ were performed on a 1 ml scale in 40 ml amber vials. Glucose oxidase (GOX) (*Aspergillus niger*, Sigma–Aldrich) generates H_2_O_2_ by the conversion of glucose to gluconic acid. The reaction mixtures contained: 15 U ml_RM_^−1^ PaDa-I, 0.1 U ml_RM_^−1^ glucose oxidase (a 10 U ml^−1^ premix in buffer was prepared and 10 µl ml_RM_^−1^ were used to setup the biotransformation) and 200 mM glucose in 100 mM potassium phosphate buffer (pH 6). Cyclohexane was added as substrate at a concentration of 100 µl ml_RM_^−1^ to start the reaction. The reactions were shaken or stirred with a magnetic stirrer bar at 200 rpm, 20 °C for 2 h. Reaction mixtures were extracted with 2 × 500 µl ethyl acetate containing 2 mM 2-decanol as internal standard and quantified by GC analysis (Supplementary Method 1). The effect of the metal catalyst on the PaDa-I/GOX reactions were investigated by adding 1 mg of metal catalysts to stirred reactions.

### Reactions using metal catalysts for in situ H_2_O_2_ production

To evaluate PaDa-I reactions using H_2_O_2_ generated in situ by metal catalysts, sets of three simultaneous reactions were performed on a 10 ml scale using 100 ml dreschel bottles. H_2_ and air from two gas reservoirs were mixed continuously. Distinct concentrations of H_2_ in air (4, 77 and 98%) were obtained by connecting the rubber tubes from the two reservoirs with a polypropylene tubing y-connector and then adjusting the gas flow from each reservoir. The concentrations of H_2_ and air were checked continuously by gas chromatography and adjusted if necessary. The gas inlets terminated below the liquid surfaces so that the gas mixture was bubbled through the stirred reaction mixtures at a rate of 30 ml min^−1^.

The reaction mixtures contained 15 U ml_RM_^−1^ PaDa-I, different concentrations (0.5, 0.25 and 0.1 mg ml_RM_^−1^) of the different metal catalyst (2.5 wt% Au–2.5 wt% Pd/TiO_2,_ 0.5 wt% Au–0.5 wt% Pd/TiO_2_, 2.5 wt% Au–2.5 wt% Pd on carbon and 5 wt% Au on carbon) in 100 mM potassium phosphate buffer (pH 6) and 100 µl ml_RM_^−1^ cyclohexane. The catalysts were weighed out directly into the glass vessels and then the buffer was added. Immediately before starting the reactions, enzyme and cyclohexane were added. The reactions were started by switching on the gas flow. The reactions were stirred with a magnetic stirrer bar at 200 rpm and ambient temperature (19.5-20.5 °C). To compensate for the evaporation of the cyclohexane, additional cyclohexane (500 µl) was added every 15 min. Product formation was monitored after 2 h (or longer times) by extracting with 2 × 2.5 ml ethyl acetate containing 2 mM 2-decanol as internal standard and subjecting samples to GC analysis (Supplementary Method 1).

For experiments performed without oxygen present in the gas mixture, the setup was slightly modified. A syringe containing substrate was connected to the tubing connected to the outlet of the glass vessel. Thinner tubing was attached to the syringe, long enough to reach about half way into the glass vessel. Reaction mixture containing buffer with or without chemical catalyst, was purged for 5 min with N_2_ gas to get rid of the dissolved oxygen. Afterwards peroxygenase was added, where needed, and the vessel was purged again with N_2_ for 5 min to get rid of the oxygen in the headspace. The vessel was then connected to H_2_ gas and 1 ml substrate was added through the syringe to start the reaction. The reactions ran for 2 h with substrate added every 15 min through the syringe.

### Reactions using a closed system

Reactions were carried out in 50 ml Radleys glass reactors stirred using a Radleys starfish multi reaction system. The reaction mixtures contained 15 U ml_RM_^−1^ PaDa-I, 0.1 mg ml_RM_^−1^ of the metal catalyst (0.5 wt% Au–0.5 wt% Pd/TiO_2_) in 100 mM potassium phosphate buffer (pH 6) and 10 mM of substrate (cyclohexane, ethylbenzene, Isophorone, propylbenzene, tetralin and styrene all sourced from Sigma–Aldrich >97%). The catalysts were weighed out directly into the glass vessels and then the buffer was added. Immediately before starting the reactions, enzyme and substrate were added. The system was then pressurized to 2 bar of 80% H_2_ 20% air from a high-pressure gas reservoir. The reactions were stirred with a magnetic stirrer bar at 250 and ambient temperature (19.5–20.5 °C). Product formation was monitored by extracting with 2 × 5 ml ethyl acetate containing 2 mM 1-decanol as internal standard and subjecting samples to GC analysis with the concentrations determined through comparisons to a known calibration factor (Supplementary Method 2). Repeat tests—indicated in Fig. 2D—show that measured reaction products agree ± 0.2 mM as determined by GC (2% of the initial reactant concentration of 10 mM) on duplicate experiments.

### Time course using GOX for in situ H_2_O_2_ production

To evaluate PaDa-I reactions using H_2_O_2_ generated in situ by GOX, sets of two simultaneous reactions were performed on a 10 ml scale using 100 ml dreschel bottles. Air was bubbled through the stirred reaction mixture at a rate of 30 ml min^−1^. The reaction mixtures contained 15 U ml_RM_^−1^ PaDa-I, 0.2 U ml_RM_^−1^ glucose oxidase and 200 mM glucose in 100 mM potassium phosphate buffer (pH 6) and 100 µl ml_RM_^−1^ cyclohexane. All the components excluding the cyclohexane was added to the glass reactor. The reactions were stirred with a magnetic stirrer bar at 200 rpm and ambient temperature (19.5–20.5 °C). The reactions were started by the addition of 1 ml cyclohexane and ran for 2 h and 8 h, substrate (500 µl) was added every 15 min. Product formation was monitored by extracting with 2 × 2.5 ml ethyl acetate containing 2 mM 2-decanol as internal standard and subjecting samples to GC analysis.

### Assay for residual peroxygenase activity

Residual activity was evaluated using the peroxygenase assay with 5-nitro-1,3-benzodioxide (NBD) as substrate and determining endpoint values. The assay mixture contained potassium phosphate buffer (100 mM, pH 7.0) (500 µl), water (380 µl), NBD (5 mM in acetonitrile) (100 µl), enzyme solution (10 µl) and hydrogen peroxide (100 mM) (10 µl). Assays were incubated at room temperature for 5 min and then stopped with 10 M NaOH (100 µl). Absorbance was read at 514 nm and activities calculated using ε_514_ = 11400 M cm^−1^. Percentage residual activity was calculated relative to activity measured after 2 min.

### Chiral GC analysis

Data were acquired on an Agilent 7890 A fitted with a Restek Rt-beta-DEXsm fused silica capillary column (30 m × 320 uM × 0.25 uM) and a flame ionisation detector (FID). Samples (5 uL) were injected at 200 °C in a 10:1 split at 26.7 mL/min total flow. The oven was equilibrated for 3 min before and after each analysis. For phenylethanol samples the oven was held at 50 °C for 1 min before heating to 200 °C at 5 °C/min and holding at 200 °C for 5 min. For other compounds, the oven was held at 100 °C for 1 min before heating to 200 °C at 3 °C/min and holding at 200 °C for 5 min.

## Supplementary information

Supplementary Information

## Data Availability

The data that support the findings of this study are available from the corresponding author upon reasonable request.
