# Supplementary material for: A chemo-enzymatic oxidation cascade to activate C–H bonds with in situ generated H2O2
Source: Nat Commun. 2019 Sep 13;10:4178. doi: 10.1038/s41467-019-12120-w (PMC6744418; doi:10.1038/s41467-019-12120-w)
Supplement: Supplementary file 1 — Supplementary Information [file 41467_2019_12120_MOESM1_ESM.pdf]

Supplementary Material for

A chemo-enzymatic oxidation cascade to activate C–H bonds with in situ generated H_2O_2

Simon J. Freakley[†1,2], Svenja Kochius[†3,4], Jacqueline van Marwijk[†3,4], Caryn Fenner[4,5], Richard J. Lewis[1], Kai Baldenius[6], Sarel S. Marais[3,4], Diederik J. Opperman[3,4], Susan T. L. Harrison[4,5], Miguel Alcalde[7], Martha S. Smit[3,4*] and Graham J. Hutchings[1*]

[1] Cardiff Catalysis Institute, School of Chemistry, Cardiff University, Main Building, Park Place, Cardiff, CF10 3AT, UK.

[2] Department of Chemistry, University of Bath, Claverton Down, Bath, BA2 7AY UK.

[3] Department of Microbial, Biochemical and Food Biotechnology, University of the Free State, Bloemfontein, South Africa.

[4] South African DST-NRF Centre of Excellence in Catalysis, c*change, University of Cape Town, Private Bag, Rondebosch 7701, Cape Town, South Africa.

[5] Centre for Bioprocess Engineering Research (CeBER), Department of Chemical Engineering, University of Cape Town, Private Bag X3, Rondebosch 7701, Cape Town, South Africa.

[6] BASF SE, RBW/OS - A 30, Carl-Bosch-Strasse 38, 67056 Ludwigshafen am Rhein, Germany.

[7] Department of Biocatalysis, Institute of Catalysis, CSIC, 28049 Madrid, Spain.

*Corresponding Authors- SmitMS@ufs.ac.za, Hutch@cardiff.ac.uk.

[†] These authors had equal contribution

Materials Included:

Supplementary Methods – 1-2
Supplementary Discussion - 1
Supplementary Figures 1-8
Supplementary Table 1

Supplementary Method 1 - Product analysis using gas chromatography

Substrate	T_R (min)	Temperature profile
cyclohexane	cyclohexanol - 7.20<br>cyclohexanone - 7.38<br>2-dodecanol -11.45	80 °C; 3.5 min hold;<br>25°C min^{-1} to 250 °C;<br>5 min hold

[a] Shimadzu GC 2010 gas chromatograph. Column VF-5ms (60 m x 0.32 mm x 0.45 mm; 0.25 µm). Injector: 240 °C. Column flow (H_2): 1.3 ml min^{-1}; Detector (FID): 280 °C

Supplementary Method 2– - Product analysis using gas chromatography

Substrate	T_R (min)	Temperature profile
cyclohexane	cyclohexanol – 7.1<br>cyclohexanone – 7.8<br>1-decanol - 10	50 °C; 1 min hold;<br>10°C min^{-1} to 220 °C;<br>1 min hold
ethylbenzene	ethylbenzene – 4.15<br>1-phenylethanol - 7<br>2-phenylethanol – 7.8<br>acetophenone – 7.2	
isophorone	isophorone – 8.1<br>4-hydroxy isophorone -10.6<br>7-hydroxy isophorone – 11.9	
propylbenzene	1-phenyl-2-propanol 8.1<br>1-phenyl-1-propanol 8.3<br>3-phenyl-1-propanol 9.4	
tetralin	tetralin – 8.5<br>tetralin-1-ol – 11.1<br>1-tetralone – 11.4	
styrene	styrene - 4.15<br>styrene oxide – 6.75	

[a] Bruker Scion 456-GC. Column HP-5 (30 m x 0.320 mm; 0.25 µm). Injector: 250 °C. Column flow (H_2): 2 ml min^{-1}; Detector (FID): 300 °C

Supplementary Discussion 1- Basic Kinetic Analysis

The reaction system used has an internal head-space volume of 90 ml – with 5.8 mmol of H_2 (vast excess) in each reaction with typically 0.1 mmol of reactant and at extended times as steady state of 0.5 mM. Initial H_2O_2 synthesis rate experiments at short time (5 min) to minimize the contribution of over hydrogenation show that the rate of H_2O_2 synthesis, rs = 8.1 mM h^{-1}. Due to the high concentrations of H_2 we assume that [H_2] and therefore the H_2O_2 synthesis rate remains constant throughout the reaction. The enzymatic reaction converts substrate and hence H_2O_2 at a rate of 0.63 mM h^{-1} based on the cyclohexane experiments reported in Figure 3A. This, once accounting for the observed steady state of H_2O_2 would mean a H_2 – Cyclohexanol selectivity of only around 8%.

Supplementary Figure 1 – H_2O_2 production by 0.5% Au- 0.5% Pd/TiO_2 (0.1 mg ml^{-1}) with various H_2 concentration in air 50 ml min^{-1} (2% (▲) 80% (■) 90% (●)) in potassium phosphate buffer 100 mM pH 6.

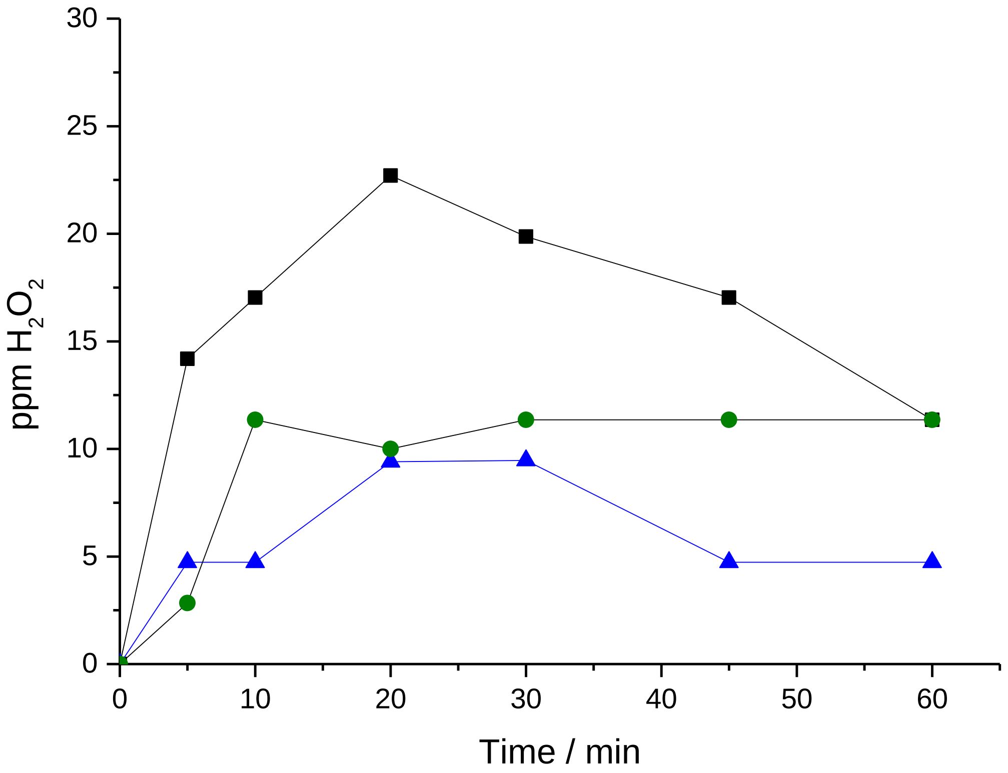

Table S1 – Cyclohexane oxidation activity in the presence of either metal catalyst (0.5 mg ml_{RM}^{-1}; 2.5% Au- 2.5% Pd/TiO_2 catalyst) or peroxygenase (15 U ml_{RM}^{-1}) when using different gas mixtures (30 ml min^{-1}) for 2 h – conditions identical to those reported for Figure 2c. Shown are the regions of the chromatograms corresponding to the retention time of cyclohexanol – in each case the test (blue) is compared to 0.01 ± 0.005 mM cyclohexanol standard (red) which represents the lowest calibration point determined at 1% of the concentration achieved in the tandem system at similar conditions – 0.9 mM cyclohexanol – reported in figure 2c.

Gas mixture	Only chemical catalyst present	Only peroxygenase present
77% H_2 in air	0.02 mM 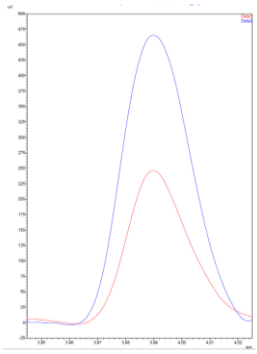	0.03 mM 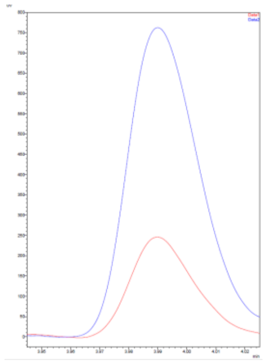
100% air	0.01 mM 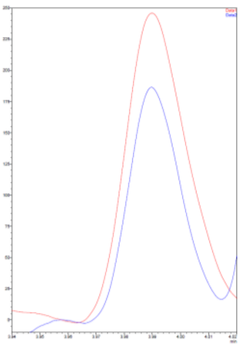	0.01 mM 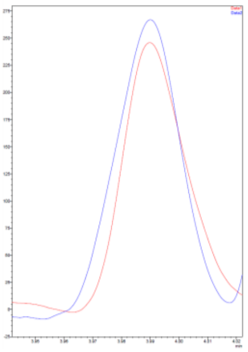

Supplementary Figure 2 – Gas Chromatograms showing cyclohexanol formation from cyclohexane with unquantifiable amounts of cyclohexanone formation.

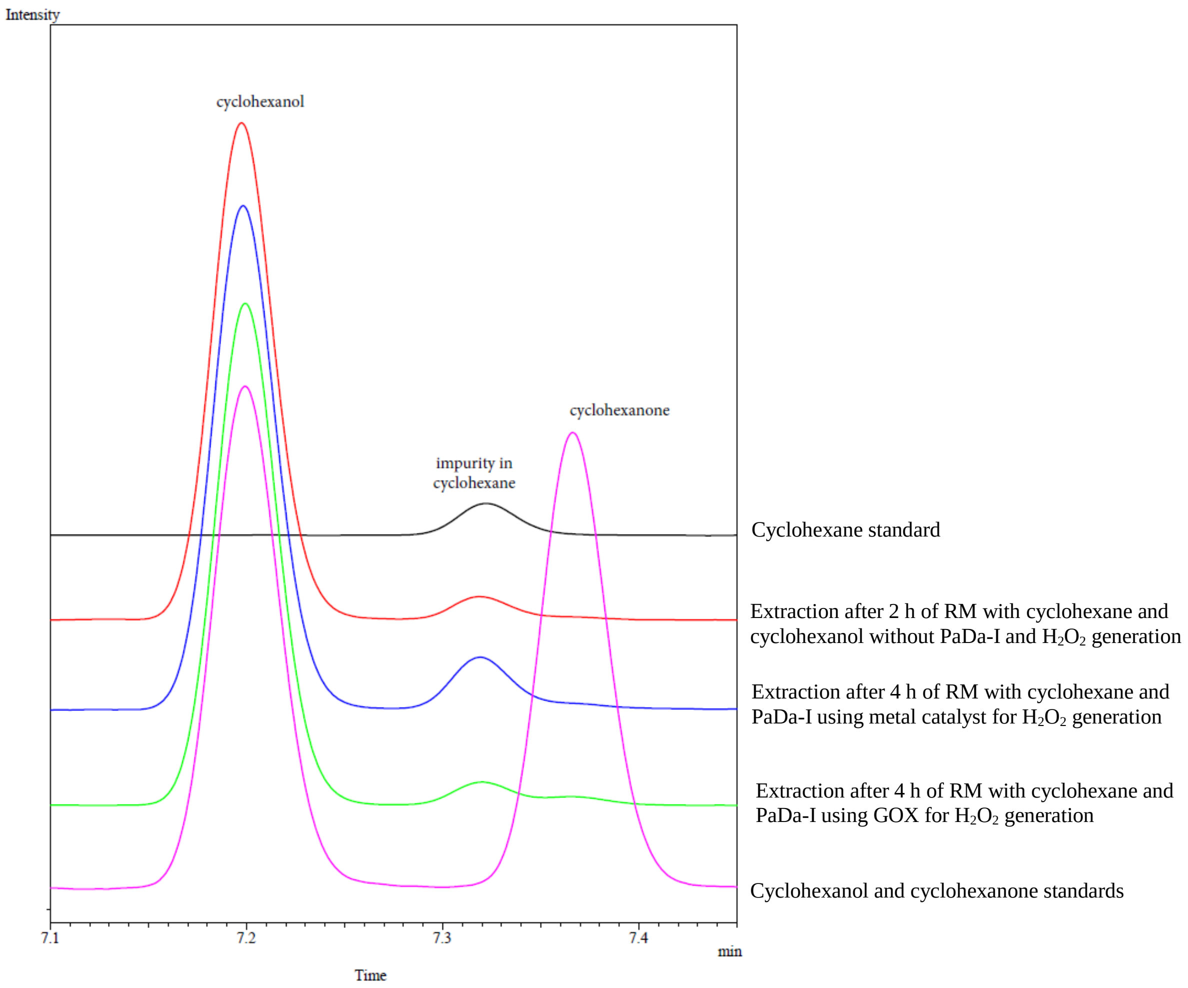

Supplementary Figure 3 – GC Chromatogram determination of enantiomeric excess for ethylbenzene hydroxylation reaction using the combination of metal and bio catalyst.

Racemic 1-phenylethanol | *S*-1-phenylethanol | 6 h ethylbenzene hydroxylation reaction

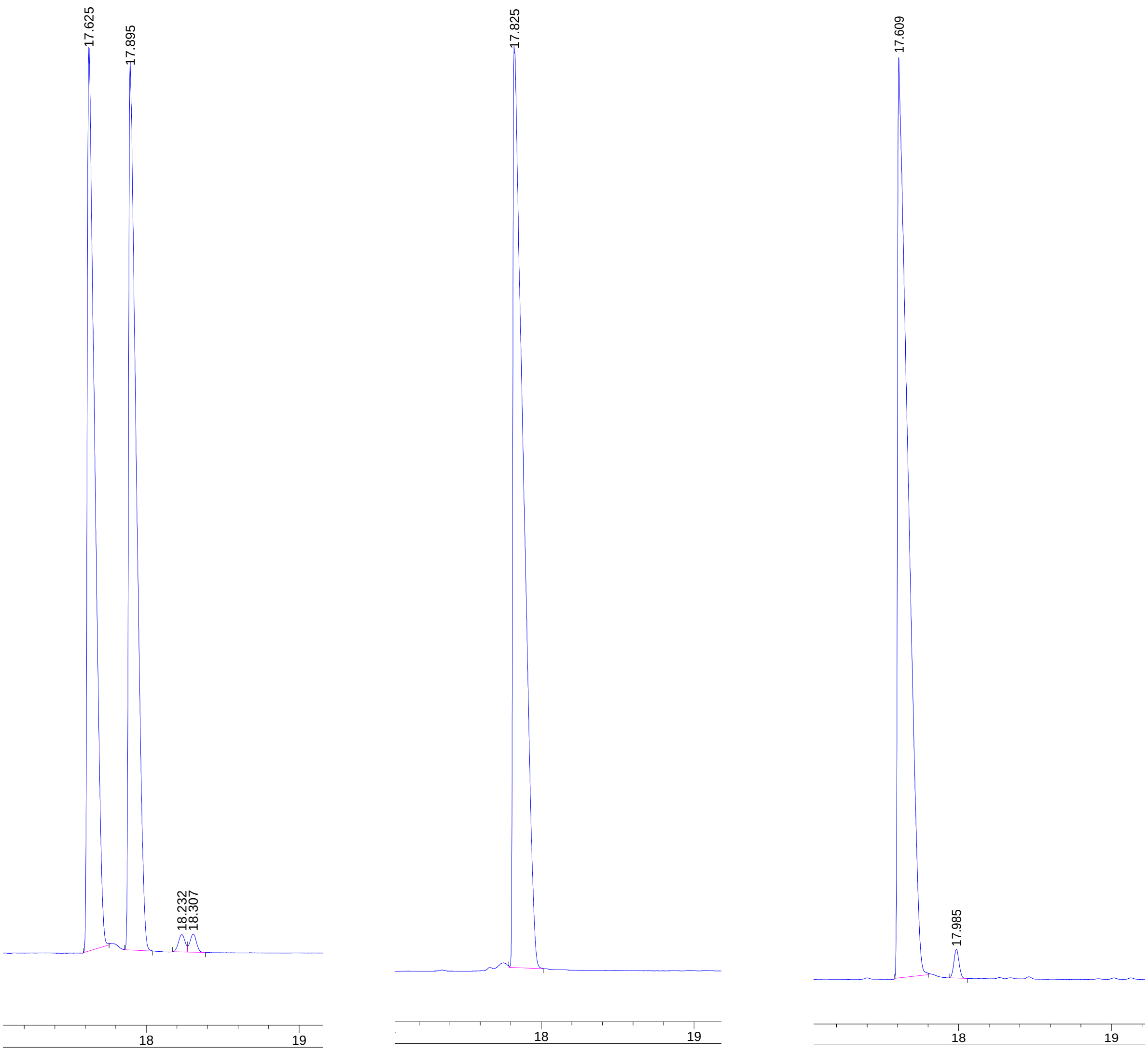

Supplementary Figure 4 –Background C-H activation activity of the 0.5% Au 0.5% Pd / TiO_2 (0.1 mg ml^{-1}) under reactive atmosphere (80% H_2 in air) towards cyclohexane, ethylbenzene and isophorone (all 10 mM starting concentration) in the absence of PaDa-I.

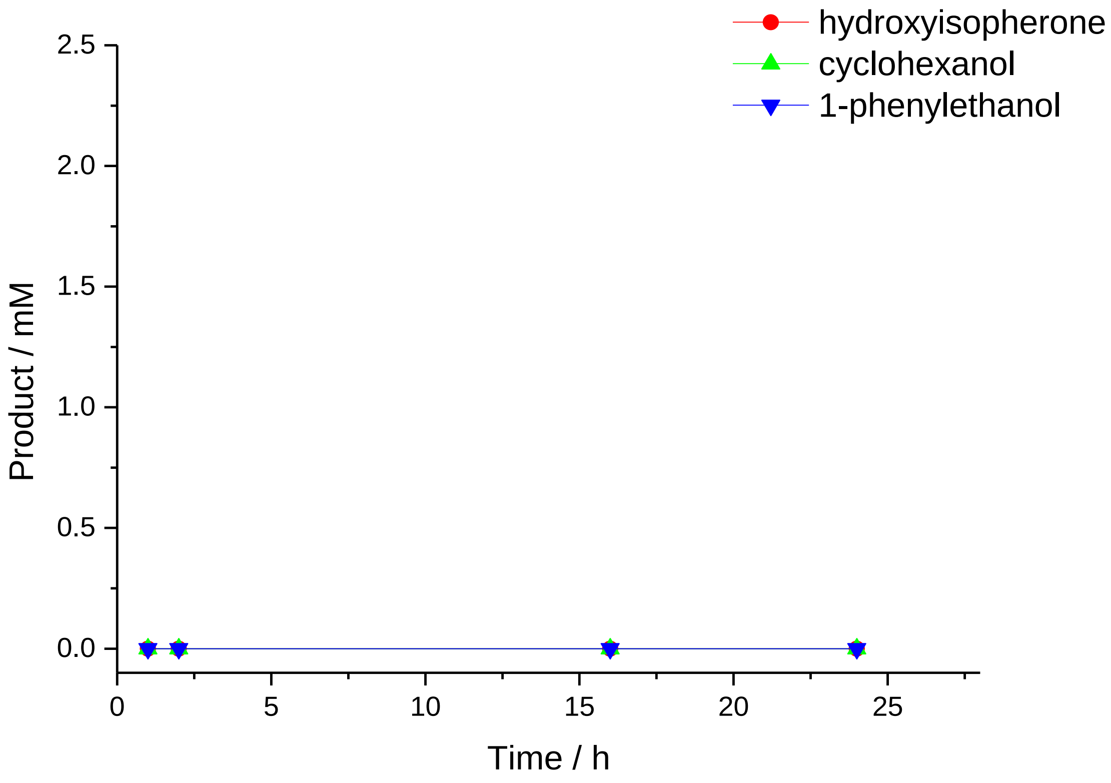

Supplementary Figure 5 –Background over oxidation activity of the 0.5% Au 0.5% Pd / TiO_2 (0.1 mg ml^{-1}) under reactive atmosphere (80% H_2 in air) towards cyclohexanol and 1-phenylethanol (both 10 mM starting concentration) in the absence of PaDa-I.

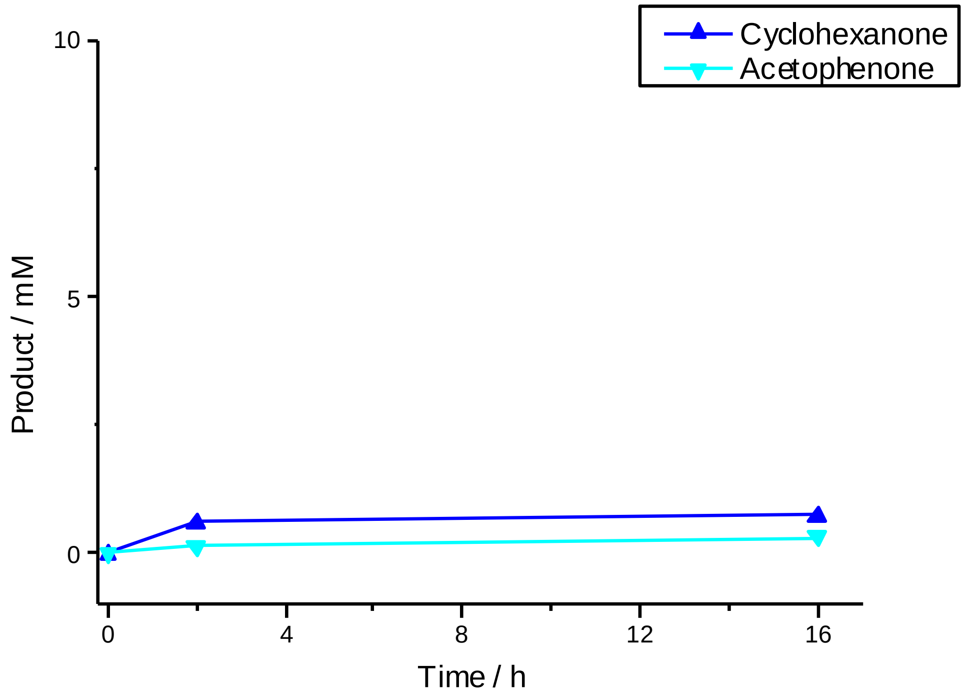

Supplementary Figure 6 –Background C-H activation activity of the 0.5% Au 0.5% Pd / TiO_2 (0.1 mg ml^{-1}) under reactive atmosphere (80% H_2 in air) towards tetralin and propylbenzene (both 10 mM starting concentration) in the absence of PaDa-I.

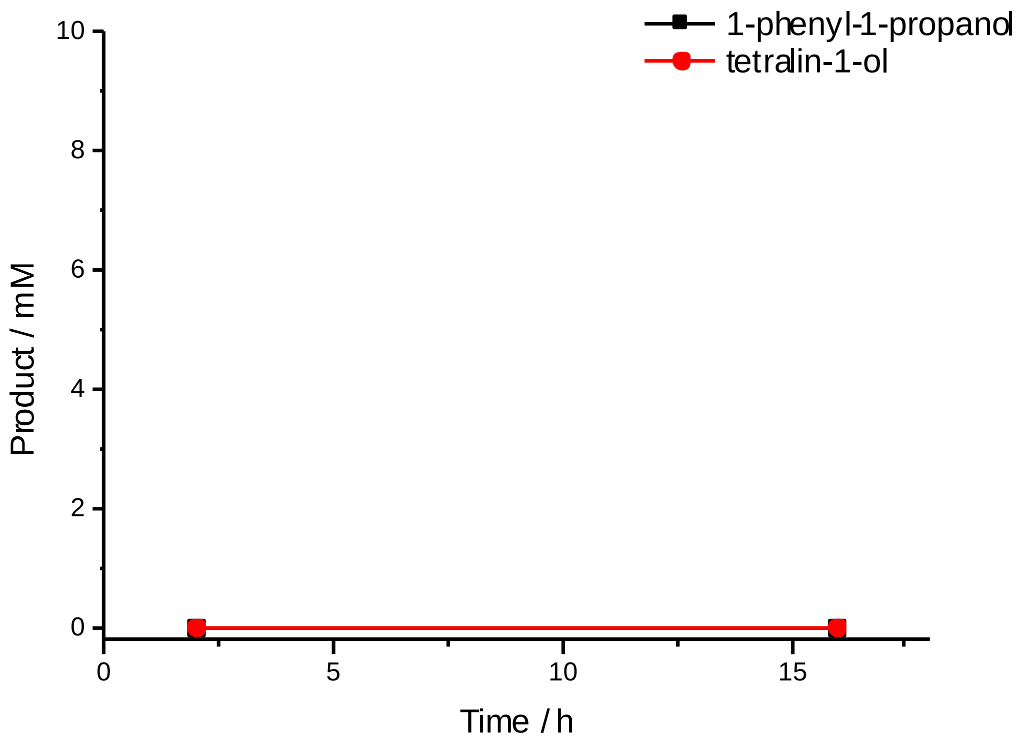

Supplementary Figure 7a – GC Chromatogram determination of enantiomeric excess for a) propylbenzene and b) tetralin hydroxylation reactions.

a)

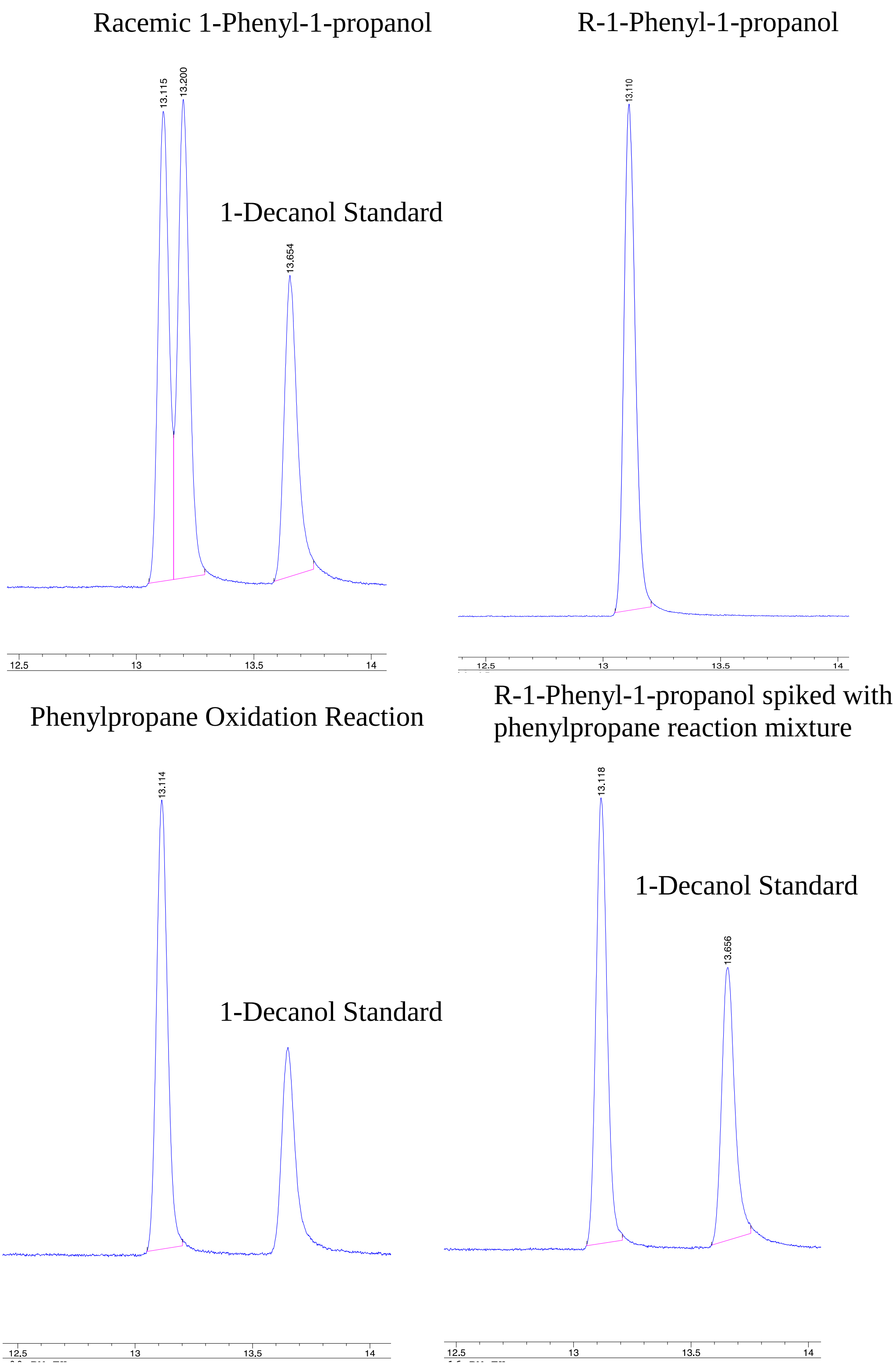

b)

Racemic tetralin-1-ol

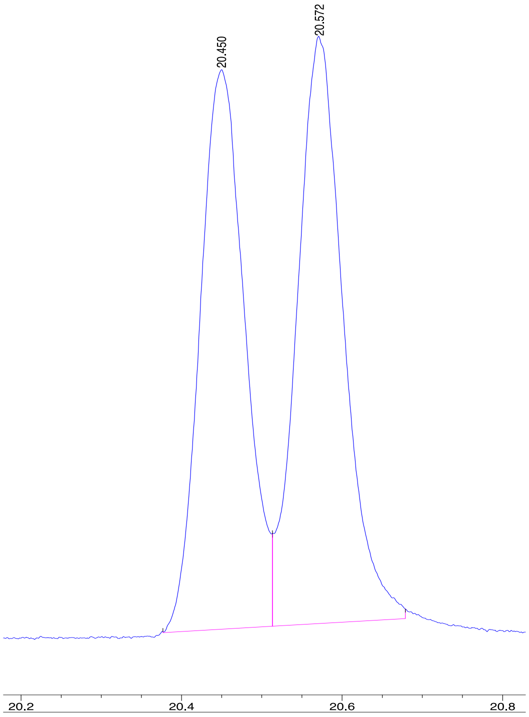

Tetralin Oxidation Reaction

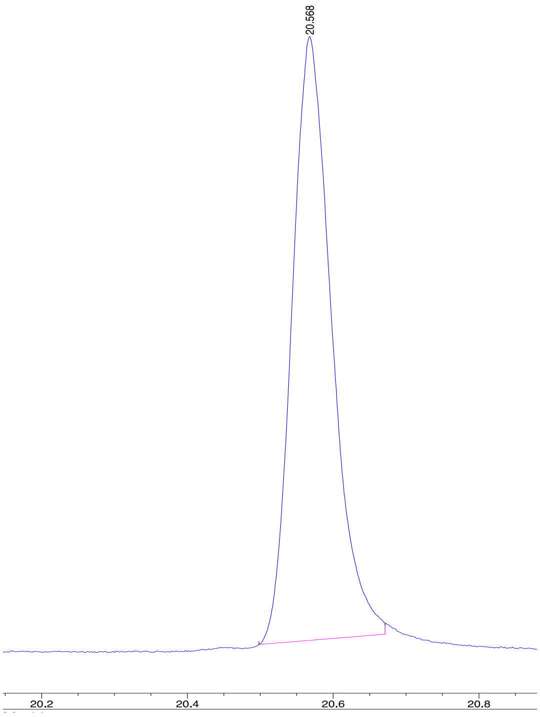

S-tetralin-1-ol spiked with reaction mixture

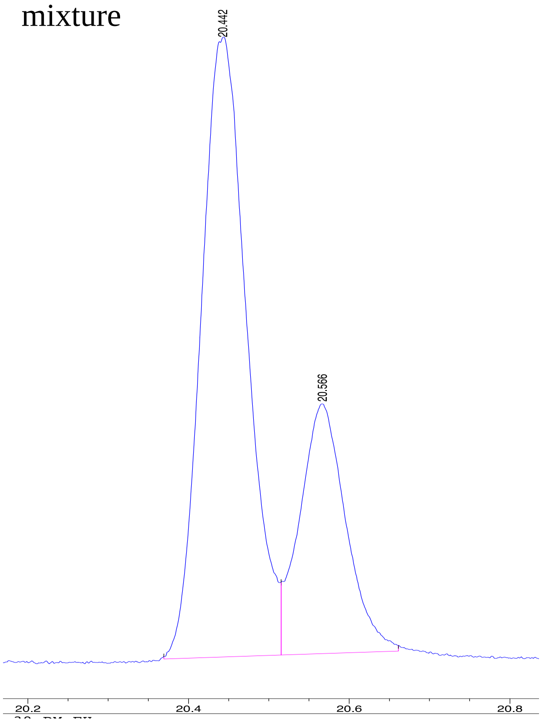

R-tetralin-1-ol spiked with reaction mixture

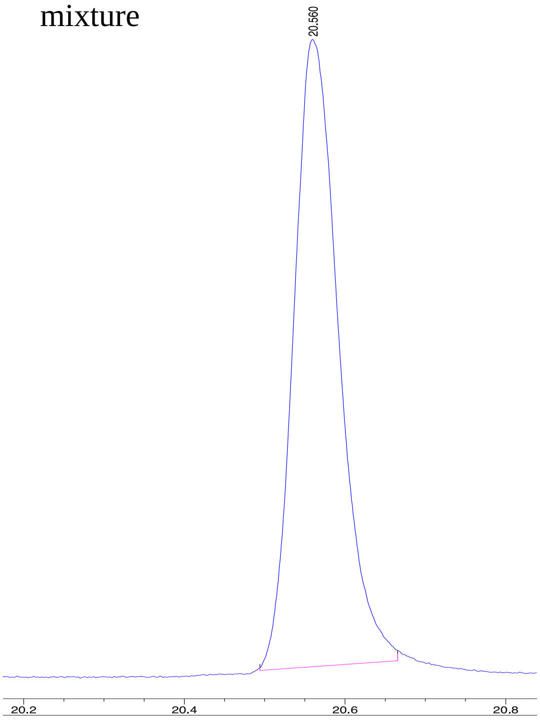

Supplementary Figure 8a – Reaction scheme combining styrene hydrogenation by the heterogeneous catalyst and hydroxylation by the tandem catalysis system **b)** Styrene hydrogenation by the 0.5% Au 0.5% Pd / TiO_2 (0.1 mg ml^{-1}) under reactive atmosphere (80% H_2 in air) in the absence of PaDa-I. **c)** Chrial analysis of the tandem catalysis reaction mixture

a)

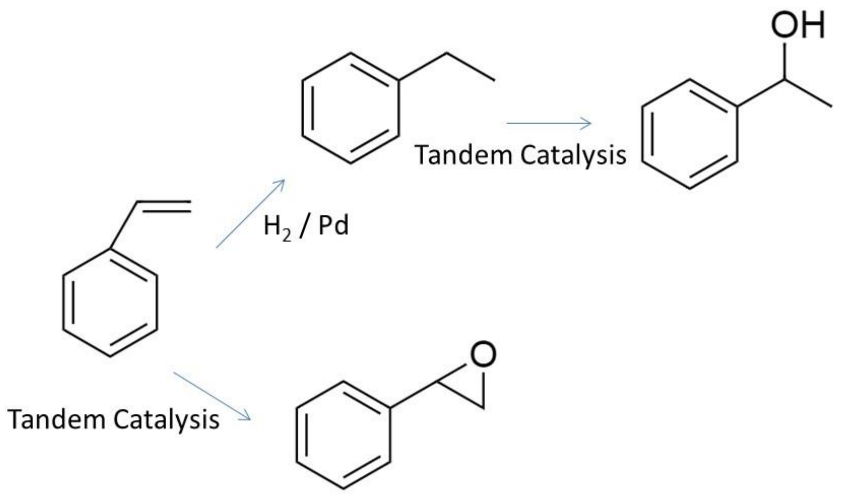

b)

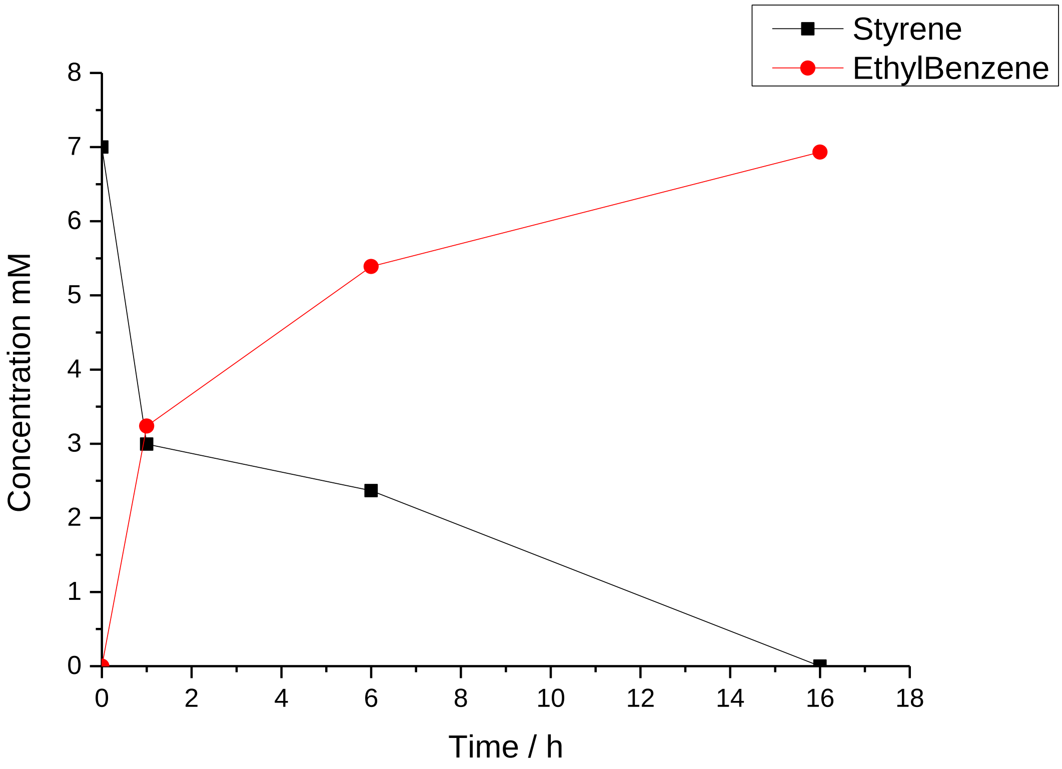

c)

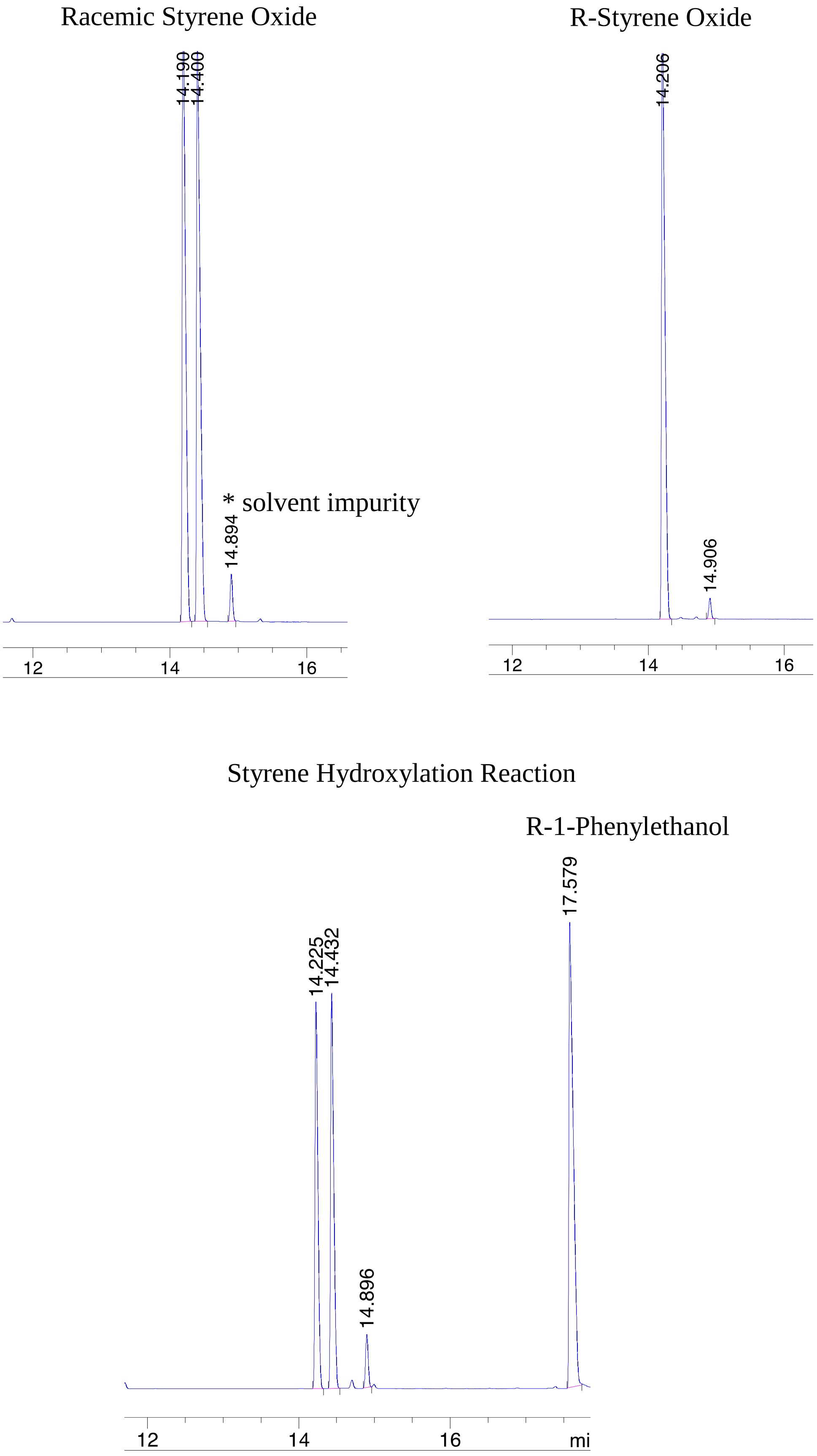
Racemic Styrene Oxide
14.199
14.400
* solvent impurity
14.894
12
14
16
R-Styrene Oxide
14.206
14.906
12
14
16
Styrene Hydroxylation Reaction
R-1-Phenylethanol
17.579
14.225
14.432
14.896
12
14
16
mi
